# Relapsed/refractory acute promyelocytic leukemia with RARA-LBD region mutation was salvaged by venetoclax: A case report

**DOI:** 10.1097/MD.0000000000028076

**Published:** 2021-12-03

**Authors:** Youli Li, Jieni Yu, Qinhong Xu, Kejie Zhang

**Affiliations:** a The Third Clinical Medical College, Fujian Medical University, Fuzhou, China; b Department of Hematology, Zhongshan Hospital, Xiamen University, Xiamen, China.

**Keywords:** acute promyelocytic leukemia, Bcl-2 inhibitors, case report, complete hematologic response, relapsed and refractory

## Abstract

**Rationale:**

Acute promyelocytic leukemia (APL) is one of the most curable cancers. However, relapse of the disease is a difficult issue in clinical practice and it remains a great challenge that patients have a poor effect of conventional treatment in the clinic. Therefore, new and more effective therapeutic measures are urgently needed. Herein, we report a case of relapsed and refractory APL harboring a RARA-LBD region mutation successfully treated with venetoclax (VEN).

**Patient concerns:**

A 37-years-old woman was admitted to our hospital with worsening spontaneous gingival bleeding and skin ecchymosis. Physical examination revealed multiple petechiae and ecchymosis in the extremities.

**Diagnoses:**

The patient was diagnosed with L-type PML-RARα-positive APL, harboring a RARA-LBD region mutation, low-risk, based on bone marrow cytology, immunophenotypic analysis by flow cytometry, karyotype analysis, and molecular analysis.

**Interventions:**

Complete remission was achieved after the first induction therapy of all-trans retinoic acid (ATRA) combined with arsenic trioxide, but relapse was observed only after 11 months. Reinduction with ATRA and arsenic trioxide combined with anthracycline failed. Therefore, we tried to provide a new treatment with the Bcl-2 inhibitor VEN orally (100 mg d1, 200 mg d2 to d18, followed by 300 mg daily continuously).

**Outcomes:**

Clinical symptoms and laboratory indicators improved rapidly with VEN treatment. A complete hematologic response was achieved with VEN-based therapy.

**Lessons:**

Related drug resistance gene monitoring should be performed canonically in relapsed and refractory APL. Some relapsed and refractory APL that failed to respond to conventional treatment were at risk of death. Bcl-2 inhibitors are expected to be an effective salvage therapy for patients with resistance to ATRA, which is worthy of further discussion.

## 1 Introduction

Acute promyelocytic leukemia (APL) is a special subtype of acute myelogenous leukemia (AML), which manifests as hemorrhage and hemocytopenia, accounting for 10% to 15% of all AML diagnoses.^[1]^ It was once considered as the most malignant form of acute leukemia with a severe bleeding tendency and a fatal course of only a few weeks. APL has now become curable with the great development of all-trans retinoic acid (ATRA). Some patients experienced recurrence, although it has a high cure rate. The poor effect of conventional treatment is a great challenge in clinical practice, and new therapeutic measures need to be developed. Relevant literature has reported that venetoclax (VEN)-based therapy is a feasible rescue therapy for relapsed and refractory nonpromyelocytic AML,^[2–4]^ but its application in APL has not yet been reported. Herein, we describe a case in which a patient with relapsed and refractory APL failed to undergo treatment with ATRA in combination with arsenic trioxide (ATO) and chemotherapy, but she achieved a complete hematologic response (CHR) with a Bcl-2 inhibitor. This is reported below.

## 2 Case presentation

**Figure 1:**
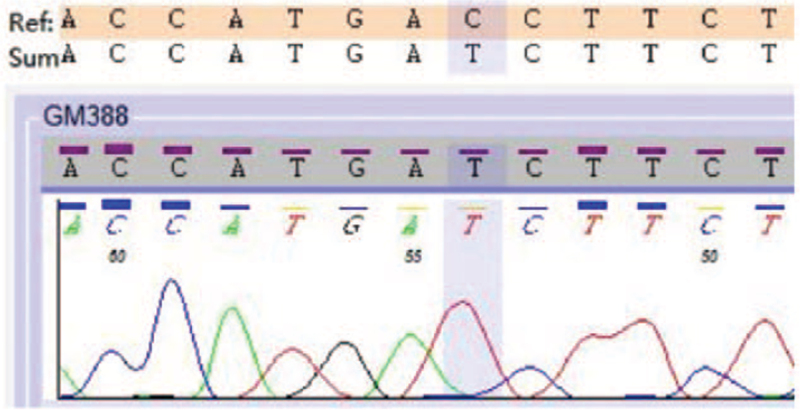
PML-RARA mutation analysis results. PML-RARA mutation analysis results were as follows: RARA-LBD area: c.854C>T; p.T285I (high mutation rate), suggesting ATRA resistance, and no ATO resistance-related gene mutation was detected.

**Figure 2:**
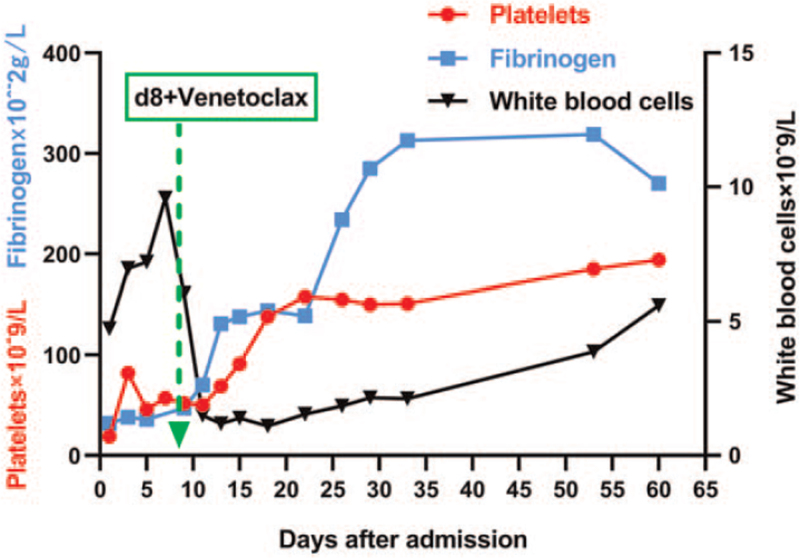
The laboratory indicators change trend of the patient. With the induction chemotherapy of ATRA combined with ATO and transfusion, fibrinogen still lasted <0.3 g/L after 8 days of treatment, and the coagulation dysfunction could not be corrected. We treated her with venetoclax orally (100 mg d1, 200 mg d2–d18, followed by 300 mg daily continuously). On the 3rd day after Bcl-2 inhibitor treatment, ecchymosis in the limbs of the patient faded obviously, and platelet and fibrinogen levels gradually increased. On the 6th day, the fibrinogen increased to 1.31 g/L and the platelet count increased to 69 × 109/L, leaving the blood transfusion treatment. On the 29th day, she achieved complete hematologic remission.

A 37-years-old woman patient visited a local hospital for worsening spontaneous gingival bleeding and skin ecchymosis in December 2018. The complete blood count results were normal in parentheses, as shown below: white blood cell: 2.96 × 10^9^/L (reference range, 4 × 10^9^/L–10 × 10^9^/L); hemoglobin: 124 g/L (120–160 g/L); platelets: 30 × 10^9^/L (100 × 10^9^/L–300 × 10^9^/L); myeloblasts 16.0%. She was diagnosed with L-type PML-RARα-positive APL, low-risk, based on bone marrow cytology, immunophenotypic analysis by flow cytometry, karyotype analysis, and molecular analysis. She achieved CHR after 1 month of induction therapy with ATRA and ATO combined with chemotherapy. Then, 3 consolidation therapies were administered to daunorubicin plus cytarabine, homoharringtonine plus cytarabine, and daunorubicin plus cytarabine successively. Maintenance therapy with ATRA and compound realgar natural indigo tablets was subsequently administered for approximately 3 cycles. She sustained CHR during this period, but no fusion gene monitoring was performed to assess whether molecular remission was achieved. Unfortunately, she experienced a relapse during the maintenance therapy in December 2019, failing to achieve CHR after double induction therapy with ATRA and ATO. She was then administered 1 dose of 40 mg idarubicin, twice that of idarubicin plus cytarabine, and 1 medium dose of 21 g cytarabine chemotherapy, all of which failed. The patient was admitted to our hospital in August 2020 for excessive fatigue and progressive skin and mucosal bleeding. On physical examination, multiple petechiae and ecchymosis were observed in the extremities, and no obvious abnormalities were observed in the rest. The complete blood count results were as follows: white blood cell: 4.73 × 10^9^/L; hemoglobin: 96 g/L; platelets: 19 × 10^9^/L; absolute neutrophil count: 0.09 × 10^9^/L (1.8 × 10^9^/L–6.3 × 10^9^/L); abnormal promyelocytes in peripheral blood account for 78%. The results of the coagulation profile were as follows: prothrombin time: 17.4 seconds (9.0–13.0 seconds); prothrombin time ratio: 1.58 (0.82–1.18); international normalized ratio: 1.60 (0.80–1.20); fibrinogen: 0.32 g/L (2.0–4.0 g/L); thrombin time: 25.9 seconds (14.0–21.0 seconds); d-dimer: 11.56 mg/L (0.00–0.55 mg/L); fibrinogen degradation products: 27.97 mg/L (0.00–5.00 mg/L). Bone marrow aspiration demonstrated relapsed APL with promyelocytes comprising 96% and L-type PML-RARα fusion gene positivity. Color ultrasonography showed right hepatic vein occlusion with possible collateral formation. After admission, we treated her with ATRA combined with ATO double-induction chemotherapy and continuous transfusion of cryoprecipitate, fresh frozen plasma, and platelets. At the same time, drug-resistance genes were tested. Fibrinogen still lasted <0.3 g/L after 8 days of treatment, and the coagulation dysfunction could not be corrected. PML-RARα mutation analysis results were as follows: RARA-LBD area: c.854C>T; p.T285I (high mutation rate), suggesting ATRA resistance, and no ATO resistance-related gene mutation was detected (Fig. 1). At one point, the patient and her family wanted to give up the treatment. Therefore, we tried to provide a new treatment with the Bcl-2 inhibitor VEN orally (100 mg d1, 200 mg d2–d18, followed by 300 mg daily continuously). On the 3rd day after the Bcl-2 inhibitor treatment, ecchymosis in the limbs of the patient faded obviously, and platelet and fibrinogen levels gradually increased. On the 6th day, the fibrinogen increased to 1.31 g/L and the platelet count increased to 69 × 10^9^/L, leaving the blood transfusion treatment. On the 29^th^ day, the patient achieved CHR (Fig. 2). During this period, the patient suffered agranulocytosis lasting for about half a month, but no fever or infection occurred. The subsequent morphology of bone marrow cells suggested that the myeloblasts plus promyelocytes accounted for 2% and the copy number of PML-RARα gene decreased by 2 logs from 78,658 to 3786, reaching CHR. Allogeneic stem cell transplantation was recommended after hematological remission, but it was not performed for financial reasons. Therefore, we administered consolidation therapy with VEN at 300 mg daily in combination with ATO. She had good adherence and tolerability. Despite agranulocytosis for half a month, she did not have a fever, tumor lytic syndrome, anemia, thrombocytopenia, or other adverse reactions, and was in continuous CHR, but never reached molecular remission with the treatment of Bcl-2 inhibitor. Unfortunately, after 4 months of maintenance treatment, the patient experienced a relapse and died 2 months later.

## 3 Discussion

APL was once the deadliest subtype of AML. The cure rate of APL has been increasing since the combination therapy of ATRA and ATO. However, the relapse rate of APL can still reach 8% to 30.5%,^[5–9]^ which has become a major factor for failed treatment and death. Some relapsed and refractory APL failed to respond to conventional treatment and were at risk of death due to severe coagulation disorders, including bleeding and thrombosis. This has forced us to search for new, safe, and effective treatment protocols. Studies have shown that Bcl-2 inhibitors can treat cancer by downregulating the expression of antiapoptotic Bcl-2 protein and promoting the apoptosis of malignant tumor cells.^[10,11]^ As an oral, potent, and selective inhibitor of Bcl-2, VEN has no decisive effect on the survival of platelets and will not cause significant reduction of platelets.^[12,13]^ Relevant clinical trials reported that treatment with VEN significantly improved the median survival time in chronic lymphocytic leukemia,^[14,15]^ AML,^[16]^ and relapse or refractory multiple myeloma,^[17]^ but no study has reported its application in APL. A part of relapsed and refractory APL develop resistance to ATRA and/or ATO.^[18–22]^ Studies have found that resistance to ATRA may be related to the mutations in RARA-LBD region.^[19,23]^ Goto et al^[24]^ and Zhu et al^[25]^ studied the mutations associated with targeted drugs for leukemia and believed that drug resistance of APL to ATO might be related to the mutation in the PML-B2 domain.

This case of relapsed and refractory APL failed to conventional treatment when the disease relapsed and had a severe tendency to face the risk of death at any time. Until she was treated with VEN, the clinical symptoms and laboratory indicators improved rapidly, leaving the risk of death and achieving a second CHR. It was well tolerated, with few side effects, using Bcl-2 inhibitors. Unfortunately, the patient eventually died after 4 months.

Molecular remission is a necessary condition for the long-term survival of APL, and the greatest deficiency in the initial treatment of this patient is that fusion gene monitoring is not standardized, which neither evaluates whether the disease has achieved molecular remission, nor can timely salvage treatment be carried out when the disease has relapsed. Therefore, related drug-resistance gene monitoring should be carried out in relapsed and refractory APL to avoid delay in treatment. If the APL resistance gene test could be performed in time when this patient relapsed, the attempt of ineffective treatment of ATRA or ATO and the irreversible consequences or even the cost of life because of delayed treatment could be avoided. The patient was well tolerated by VEN and the obtained CHR suggested that the significant effect of VEN on this patient, as well as Bcl-2 inhibitor, is expected to be an effective salvage therapy for patients with resistance to ATRA, which is worthy of further discussion. Meanwhile, Bcl-2 inhibitor monotherapy cannot maintain long-term remission, but can be used as an effective bridging therapy. Hematopoietic stem cell transplantation should be performed as soon as possible after obtaining CHR, so that patients hope for a cure again.

## 4 Conclusion

Bcl-2 inhibitor-based therapy is expected to be an effective salvage therapy for relapsed and refractory APL harboring RARA-LBD region mutation, which is worthy of further prospective studies.

***Data access statement***

All relevant data are within the paper and its Supporting Information files. The laboratory indicators data are provide in the Supplementary Files ().

## Author contributions

**Conceptualization:** Kejie Zhang.

**Date curation:** Youli Li.

**Funding acquisition:** Kejie Zhang.

**Investigation:** Youli Li.

**Methodology:** Kejie Zhang.

**Project administration:** Youli Li.

**Resources:** Jieni Yu, Qinhong Xu, Kejie Zhang.

**Supervision:** Jieni Yu, Qinhong Xu.

**Visualization:** Jieni Yu, Qinhong Xu.

**Writing – original draft:** Youli Li.

**Writing – review & editing:** Kejie Zhang.

## Supplementary Material

Supplemental Digital Content
