# Supplementary material for: Relapsed/refractory acute promyelocytic leukemia with RARA-LBD region mutation was salvaged by venetoclax: A case report
Source: Medicine (Baltimore). 2021 Dec 3;100(48):e28076. doi: 10.1097/MD.0000000000028076 (PMC9191359; doi:10.1097/MD.0000000000028076)
Supplement: Supplemental Digital Content [file medi-100-e28076-s001.docx]

| Table 1. The laboratory indicators data | | | |
| --- | --- | --- | --- |
| Days after admission | Platelets×10ˆ9/L | Fibrinogen  g/L❋ | White blood cells  ×10ˆ9/L |
| 1 | 19 | 0.32 | 4.73 |
| 3 | 82 | 0.38 | 6.96 |
| 4 | NA | <0.30 | NA |
| 5 | 46 | 0.36 | 7.20 |
| 7 | 57 | <0.30 | 9.59 |
| 9 | 52 | 0.47 | 6.04 |
| 11 | 50 | 0.70 | 1.49 |
| 13 | 69 | 1.31 | 1.19 |
| 15 | 91 | 1.38 | 1.39 |
| 18 | 138 | 1.44 | 1.10 |
| 22 | 158 | 1.39 | 1.54 |
| 26 | 155 | 2.34 | 1.85 |
| 29 | 150 | 2.85 | 2.15 |
| 33 | 151 | 3.13 | 2.12 |
| 53 | 185 | 3.19 | 3.87 |
| 60 | 194 | 2.70 | 5.60 |
|  |  |  |  |
| ❋ To make the picture more intuitive, we artificially made the fibrinogen 100 times bigger in the figure 2. | | | |
